# Effect of Pre-Incisional Intravenous Paracetamol–Ibuprofen on Post-Laparoscopic Shoulder Pain After Laparoscopic Cholecystectomy: A Double-Blind Randomised Controlled Trial

**DOI:** 10.3390/jcm15187249

**Published:** 2026-09-18

**Authors:** Seung-Wan Hong, Seong-Hyop Kim

**Affiliations:** 1 Department of Anesthesiology and Pain Medicine, Konkuk University Medical Center, Konkuk University School of Medicine, Seoul 05030, Republic of Korea; 2 Department of Infection and Immunology, Konkuk University School of Medicine, Seoul 05030, Republic of Korea; 3 Department of Medicine, Institute of Biomedical Science and Technology, Konkuk University School of Medicine, Seoul 05030, Republic of Korea

**Keywords:** paracetamol, ibuprofen, shoulder pain, laparoscopic cholecystectomy

## Abstract

**Background**: This study was designed to evaluate the analgesic effect of pre-incisional intravenous administration of a fixed-dose combination of paracetamol and ibuprofen (Maxigesic^®^; Kyungbo Pharmaceutical, Seoul, Republic of Korea), with particular attention to post-laparoscopic shoulder pain (PLSP), in patients undergoing laparoscopic cholecystectomy (LC). **Methods**: Patients undergoing LC were enrolled in a double-blind randomised controlled trial and assigned in a 1:1 ratio to receive either normal saline (control group) or Maxigesic^®^ (study group). According to group allocation, normal saline or Maxigesic^®^ was administered intravenously over 15 min before surgical incision. PLSP, pain severity assessed using a visual analogue scale, and the proportion of patients requiring rescue analgesia for postoperative pain were evaluated during the post-anaesthetic care unit (PACU) stay (T_PACU_), from PACU discharge to 24 h after discharge (T_24h_), and from 24 to 48 h after PACU discharge (T_48h_). **Results**: The two groups had comparable demographic characteristics. Abdominal-pain scores were significantly lower in the study group during the early postoperative period. The incidence of PLSP was significantly lower in the study group at T_PACU_ and T_24h_ (T_PACU_: 16/58 in the control group vs. 5/58 in the study group, *p* = 0.014; T_24h_: 23/58 vs. 9/58, *p* = 0.006), with an absolute risk reduction of 24.1 percentage points (95% confidence interval, 8.5–39.8) during T_24h_. Among patients who developed PLSP, shoulder-pain severity did not differ significantly between the groups. A significantly lower proportion of patients in the study group required rescue analgesia for postoperative pain. No patient in either group reported PLSP at T_48h_. There were no significant differences between the two groups in postoperative nausea and vomiting. **Conclusions**: Pre-incisional intravenous administration of a fixed-dose paracetamol–ibuprofen combination was associated with a lower incidence of PLSP and a lower proportion of patients requiring rescue analgesia in patients undergoing LC, although shoulder-pain severity among patients who developed PLSP was not significantly reduced.

## 1. Introduction

With ongoing technical advances, minimally invasive surgical procedures, rather than open surgical approaches, have become increasingly common in clinical practice. Laparoscopic cholecystectomy (LC) is one such procedure. Compared with open cholecystectomy, LC offers less postoperative pain and greater patient comfort because of its minimally invasive approach. However, patients undergoing LC may still experience abdominal incisional or visceral pain [1]. In addition, laparoscopy-related shoulder pain occurs frequently. Post-laparoscopic shoulder pain (PLSP) is sometimes more uncomfortable than abdominal incisional or visceral pain [2]. Numerous trials have been conducted to prevent postoperative pain, including PLSP, after LC and have demonstrated beneficial effects; however, complete prevention remains difficult [3,4]. Recently, an analgesic combining two agents with different analgesic properties—paracetamol and ibuprofen, a non-steroidal anti-inflammatory drug (NSAID)—has become available (Maxigesic^®^; Kyungbo Pharmaceutical, Seoul, Republic of Korea). This combination has demonstrated analgesic effects in various surgical procedures [5,6]. However, its analgesic effect when administered before surgical incision has not been evaluated for postoperative pain, including PLSP, in patients undergoing LC.

We hypothesised that pre-incisional intravenous administration of a fixed-dose combination of paracetamol and ibuprofen (Maxigesic^®^) would improve postoperative pain outcomes, including PLSP, in patients undergoing LC. Given that paracetamol combined with an NSAID is already an established component of multimodal analgesia, this study specifically aimed to evaluate whether a single pre-incisional intravenous fixed-dose combination could reduce PLSP, a distinct and clinically relevant component of postoperative pain after LC.

## 2. Materials and Methods

### 2.1. Study Population

Participants were recruited from December 2025 to June 2026, with the final participant enrolled on 27 June 2026. After obtaining approval from the Institutional Review Board of Konkuk University Medical Center, Seoul, Republic of Korea (Reference No. KUH 2025-01-001; date of approval, 13 February 2025) and written informed consent from patients, individuals with American Society of Anesthesiologists (ASA) physical status I and a body mass index (BMI) < 30 kg/m^2^ undergoing LC were enrolled. The study was registered with the Clinical Research Information Service of the Korea Centers for Disease Control and Prevention, Ministry of Health and Welfare (KCT0011108; date of registration, 6 November 2025; http://cris.nih.go.kr). Patients were excluded if any of the following criteria were present: urgent or emergent surgery, regular use of acetaminophen or NSAIDs, hypersensitivity to acetaminophen or NSAIDs, relevant hepatic or renal dysfunction, gastrointestinal ulcer or bleeding, history of asthma, previous abdominal surgery, concurrent surgery, or pregnancy. Before induction of anaesthesia, the patients were randomly assigned in a 1:1 ratio to receive either normal saline (control group) or Maxigesic^®^ (study group). The random allocation sequence was generated by an independent statistical support unit using permuted block randomisation with variable block sizes of 4 and 6, without stratification. The block sizes and allocation sequence were concealed from the investigators responsible for participant enrolment. Allocation assignments were placed in sequentially numbered, opaque, securely sealed envelopes. After definitive enrolment of each participant, the next envelope in the sequence was opened only by a separate anaesthesia nurse responsible for study-drug preparation.

The anaesthesia nurse responsible for study-drug preparation was aware of the treatment allocation and prepared the assigned study solution accordingly. This nurse had no subsequent role in anaesthetic management, postoperative patient care, or outcome assessment. Participants, attending anaesthesiologists, surgeons, post-anaesthetic care unit (PACU) and ward staff, and study investigators responsible for outcome assessment remained blinded to treatment allocation. Treatment allocation remained concealed from the investigators until completion of outcome collection.

### 2.2. Anaesthesia

General anaesthesia was standardised in all patients. Anaesthesia was induced with intravenous propofol 2 mg/kg and remifentanil using target-controlled infusion according to the Minto model at a target plasma concentration of 5 ng/mL [7]. Rocuronium 0.6 mg/kg was administered to facilitate tracheal intubation. Anaesthesia was maintained with sevoflurane, titrated to maintain bispectral index values between 50 and 60, while remifentanil was maintained at a target plasma concentration of 5 ng/mL until the end of surgery. Patients were ventilated with 40% inspired oxygen using a tidal volume of 6 mL/kg ideal body weight without positive end-expiratory pressure (PEEP), and the respiratory rate was adjusted to maintain end-tidal carbon dioxide between 35 and 40 mmHg. Additional rocuronium was administered as required. Ramosetron 0.3 mg was administered for prophylaxis of postoperative nausea and vomiting (PONV). Haemodynamic variables were managed using vasoactive agents as clinically indicated. At the end of anaesthesia, residual neuromuscular blockade was reversed with sugammadex, and patients were transferred to the post-anaesthetic care unit.

### 2.3. Intervention

Before induction of anaesthesia, normal saline for the control group and Maxigesic^®^ for the study group were prepared according to group allocation by a separate anaesthesia nurse who was aware of treatment allocation and was not involved in subsequent patient care or outcome assessment. Normal saline 100 mL for the control group or Maxigesic^®^ (paracetamol 1000 mg and ibuprofen 300 mg/100 mL) for the study group was transferred into identical 100 mL infusion bags with no labelling.

The prepared infusion was administered intravenously over 15 min, beginning approximately 15 min before surgical incision, and was completed before incision.

### 2.4. LC

LC under general anaesthesia was performed by experienced surgeons using a standardised four-port technique. After induction of anaesthesia, patients were placed in the reverse Trendelenburg position with left tilt. Pneumoperitoneum was established using CO_2_ insufflation via a Veress needle inserted at the umbilicus, and intra-abdominal pressure was maintained at 12 mmHg throughout the procedure. After creation of the pneumoperitoneum, a 10 mm laparoscope was introduced through the umbilical port, and three additional 5 mm working trocars were inserted in the epigastric and right subcostal regions. The gallbladder was dissected from the liver bed after identification and clipping of the cystic duct and artery. The resected gallbladder was extracted through the umbilical port. Surgical time was defined as the interval from skin incision to skin closure.

### 2.5. Measurements

After arrival in the operating room and completion of routine non-invasive monitoring, mean blood pressure (mBP), heart rate (HR), and bispectral index (BIS) were assessed at baseline (T_0_). mBP, HR, and BIS were subsequently measured every 5 min (T_x_) until the end of LC and transfer to the PACU.

Postoperative pain, including abdominal and shoulder pain, was assessed using a visual analogue scale (VAS) ranging from 0 (no pain) to 100 (worst pain imaginable) during the following intervals: from arrival in the PACU to discharge from the PACU (T_PACU_), from discharge from the PACU to 24 h after PACU discharge (T_24h_), and from 24 h after PACU discharge to 48 h after PACU discharge (T_48h_). In the PACU, abdominal and shoulder pain were assessed at rest by a study investigator immediately after arrival and again before discharge. After PACU discharge, shoulder pain was assessed at rest through repeated patient interviews performed by study investigators at approximately 6 h intervals. At each assessment, patients were asked separately about the presence of PLSP and the intensity of shoulder pain using the VAS. After PACU discharge, abdominal-pain VAS values were obtained from routine ward nursing records. For each predefined postoperative interval, the highest recorded VAS value was used for analysis. Movement-evoked pain was not assessed separately. For shoulder pain, the occurrence of PLSP and its severity were evaluated separately. PLSP severity was assessed using the maximum shoulder-pain VAS only among patients who experienced PLSP during the corresponding interval.

Rescue analgesia in the PACU was administered only after the initial postoperative pain assessment. Ketorolac 30 mg was administered intravenously on demand as the first-line rescue analgesic for postoperative pain. Fentanyl 25 µg was administered intravenously on demand as the second-line rescue analgesic and was given only after confirmation of insufficient pain relief following ketorolac administration. If rescue analgesia was required more than 6 h after ketorolac administration, ketorolac 30 mg was administered intravenously again. PONV was assessed using a four-point ordinal scale (0 = none, 1 = nausea, 2 = retching, 3 = vomiting) [8]. Assessment was performed concurrently with postoperative pain evaluation. The severity of PONV during T_24h_ and T_48h_ was further evaluated using the Rhodes index (Rhodes_24h_ and Rhodes_48h_). This index quantifies PONV severity on a numerical scale of 0–32 and incorporates both subjective components (severity) and objective components (presence or absence of nausea, retching, and vomiting, and the frequency of these events) [9].

Dexamethasone 5 mg was administered intravenously on demand as the first-line rescue treatment for PONV. Metoclopramide 10 mg was administered intravenously on demand as the second-line rescue treatment. After discharge from the PACU, ramosetron 0.3 mg was administered intravenously on demand as rescue therapy for PONV in accordance with institutional practice.

### 2.6. Statistics

The primary outcome was the incidence of PLSP during the T_24h_ interval. The maximum shoulder-pain VAS during the T_24h_ interval was a secondary outcome. In a pilot study of 10 patients per group, PLSP during T_24h_ occurred in five patients in the control group and two patients in the study group, corresponding to incidences of 50% and 20%, respectively. Among patients who developed PLSP in the pilot study, the maximum shoulder-pain VAS during T_24h_ was 58 ± 8 in the control group and 47 ± 7 in the study group. Using G*Power software version 3.1.9.7 (Universität Kiel, Kiel, Germany), a sample size of 58 patients per group was calculated for the primary outcome based on the difference in PLSP incidence, assuming a power of 0.90 and an α level of 0.05. For the secondary shoulder-pain VAS outcome, 11 patients per group were estimated to be required using the same power and α level. Because the larger sample size was determined by the primary outcome, 58 patients per group (116 patients in total) were required. Allowing for a 10% dropout rate, the planned enrolment was 128 patients.

Statistical analyses were performed using SPSS for Windows version 27.0 (IBM Corp., Armonk, NY, USA). Categorical variables were compared using the chi-square test or Fisher’s exact test, as appropriate. Continuous variables were presented as mean ± standard deviation or median [interquartile range], depending on the data distribution, and were compared using the independent *t*-test or Mann–Whitney *U* test, as appropriate. Repeated intraoperative haemodynamic measurements were analysed using linear mixed-effects models, with group, time, and the group-by-time interaction included as fixed effects and an autoregressive [AR(1)] covariance structure used to account for within-patient correlation over time.

Postoperative outcomes, including pain scores and PONV measures, were analysed separately for each predefined time interval (T_PACU_, T_24h_, and T_48h_), using the maximum value recorded within each interval. PLSP incidence was analysed in the entire study population. In a post hoc reanalysis of the prespecified secondary outcome, shoulder-pain severity was analysed only among patients who developed PLSP during the corresponding interval. Abdominal-pain VAS was analysed in the entire study population. For outcomes assessed repeatedly across the three postoperative intervals, a Bonferroni-adjusted significance threshold of α = 0.05/3 (0.0167) was applied to interval-wise comparisons. The proportion of patients requiring rescue analgesics was analysed as a binary outcome, defined as at least one administration during the corresponding interval. Except where Bonferroni adjustment was applied, a two-sided *p* value of <0.05 was considered statistically significant. All randomised participants received the assigned intervention and were analysed according to their assigned group. There were no missing data for the prespecified postoperative outcomes, and no imputation was required.

## 3. Results

**Figure 1:**
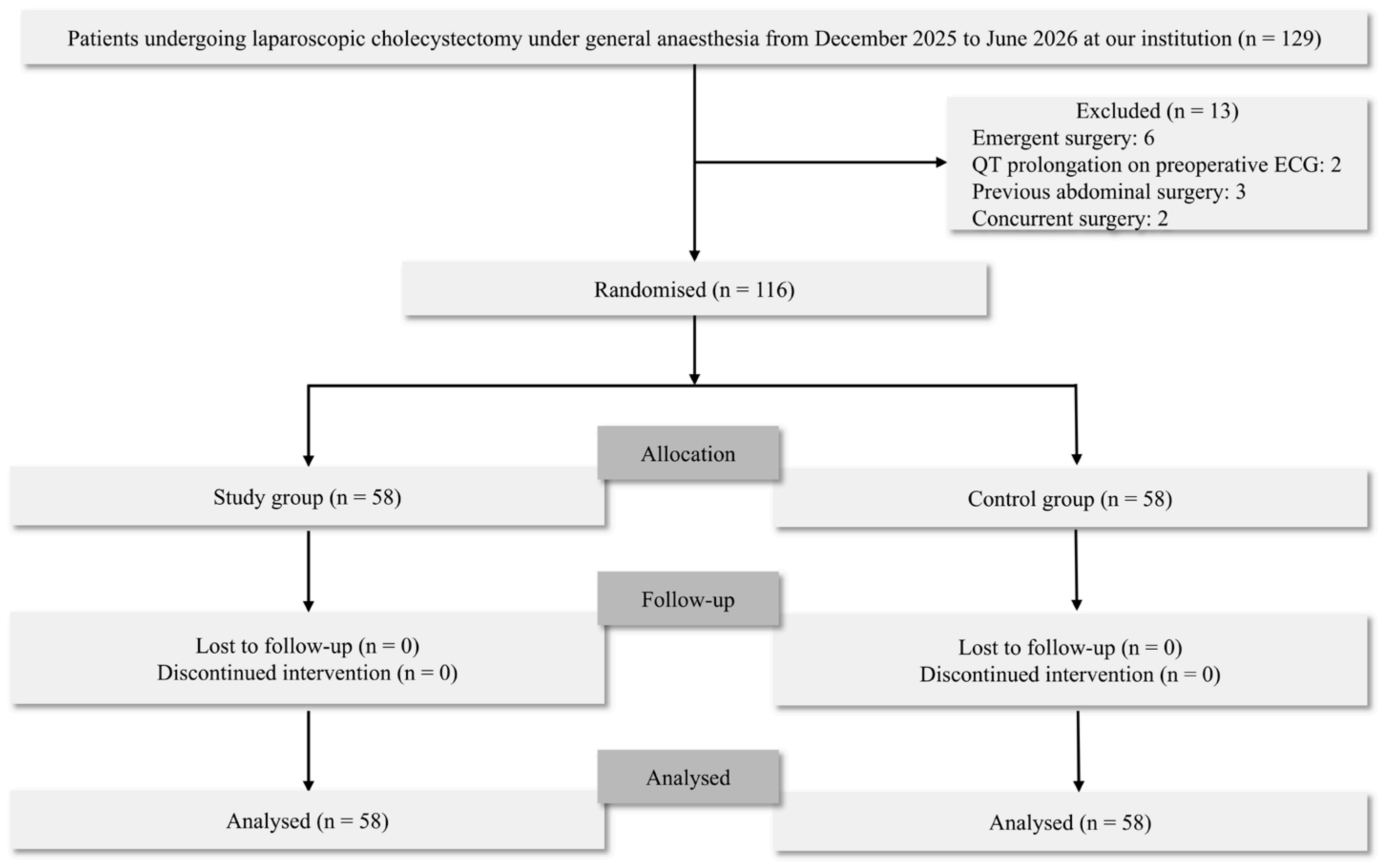
CONSORT flow diagram. Abbreviation: ECG, electrocardiogram.

In total, 129 patients were assessed for eligibility, from December 2025 to June 2026. Of these, 13 patients were excluded for the following reasons: emergent surgery (*n* = 6), QT prolongation on preoperative electrocardiography (*n* = 2), previous abdominal surgery (*n* = 3), and concurrent surgery (*n* = 2). Consequently, 116 patients were included in the final analysis, with 58 patients allocated to each group (Figure 1).

The two groups had comparable demographic and perioperative characteristics (Table 1).

Intraoperative mBP, HR, and BIS showed no significant group-by-time interactions between the two groups (mBP, *p* = 0.206; HR, *p* = 0.881; BIS, *p* = 0.764) (Figure S2).

Postoperative abdominal pain was significantly less severe in the study group at T_PACU_ and T_24h_ (T_PACU_: 40 [30–50] in the control group vs. 30 [20–40] in the study group, *p* < 0.001; T_24h_: 30 [30–40] in the control group vs. 20 [20–30] in the study group, *p* < 0.001) (Table 2). No significant difference was observed between the groups at T_48h_ (Table 2).

The study group had a significantly lower incidence of PLSP at T_PACU_ and T_24h_ (T_PACU_: 16/58 in the control group vs. 5/58 in the study group, *p* = 0.014; T_24h_: 23/58 in the control group vs. 9/58 in the study group, *p* = 0.006). During T_24h_, this corresponded to an absolute risk reduction of 24.1 percentage points (95% CI, 8.5–39.8) and a relative risk of 0.39 (95% CI, 0.20–0.77) (Table 2). Among patients who developed PLSP, shoulder-pain severity did not differ significantly between the groups. At T_PACU_, the maximum shoulder-pain VAS was 30 [20–30] in the control group (*n* = 16) and 30 [25–35] in the study group (*n* = 5) (*p* = 0.577). At T_24h_, the corresponding values were 20 [20–30] (*n* = 23) and 20 [20–30] (*n* = 9), respectively (*p* = 0.426) (Table 2). No patient in either group reported PLSP at T_48h_.

A significantly lower proportion of patients in the study group required rescue analgesics at T_PACU_ (ketorolac: 36/58 in the control group vs. 17/58 in the study group, *p* < 0.001; fentanyl: 26/58 vs. 9/58, *p* < 0.001) and during T_24h_ (ketorolac: 20/58 vs. 5/58, *p* < 0.001; fentanyl: 14/58 vs. 3/58, *p* = 0.007) (Table 2). There was no significant between-group difference in the proportion of patients requiring rescue analgesia during T_48h_ (Table 2).

No significant difference in PONV was observed between the two groups at any assessment time point. Similarly, there was no significant difference in the requirement for rescue treatment for PONV (Table 2).

## 4. Discussion

The principal finding of this study was that pre-incisional intravenous administration of a fixed-dose combination of paracetamol and ibuprofen reduced the incidence of PLSP and the proportion of patients requiring rescue analgesia during the early postoperative period after LC. However, among patients who developed PLSP, shoulder-pain severity did not differ significantly between the groups. Thus, the observed benefit appears to relate primarily to reducing the occurrence of PLSP rather than attenuating its severity once established.

PLSP is a distinct component of postoperative pain after laparoscopic surgery and is thought to result from several mechanisms, including diaphragmatic irritation and phrenic nerve stimulation associated with pneumoperitoneum, residual CO_2_, surgical manipulation, and inflammatory responses [3,10]. Various preventive approaches have therefore been investigated, including optimisation of pneumoperitoneum, removal of residual CO_2_, ventilatory strategies, local interventions, and perioperative analgesia [11,12]. In the present study, pneumoperitoneum was maintained at 12 mmHg and mechanical ventilation was performed with a tidal volume of 6 mL/kg ideal body weight without PEEP. Within this standardised perioperative setting, the present findings suggest that pharmacological intervention with a pre-incisional fixed-dose paracetamol–ibuprofen combination may provide an additional strategy for reducing PLSP.

Previous studies have shown that intravenous paracetamol or ibuprofen can reduce PLSP after LC [13,14,15]. Erdi et al. reported that both intravenous acetaminophen and ibuprofen reduced PLSP, whereas other studies demonstrated beneficial effects of perioperative intravenous paracetamol [13,14,15]. Combination analgesic regimens have also provided better postoperative analgesia than paracetamol monotherapy [16]. These findings support the use of analgesics with complementary mechanisms as part of multimodal perioperative analgesia. Paracetamol predominantly exerts central antinociceptive effects, whereas ibuprofen inhibits cyclooxygenase-mediated prostaglandin synthesis and provides peripheral anti-inflammatory and analgesic effects. Because both nociceptive and inflammatory mechanisms may contribute to PLSP, their combined use is biologically plausible. Nevertheless, the present trial compared the fixed-dose combination only with placebo and did not include separate paracetamol or ibuprofen groups. Therefore, the findings cannot establish pharmacological synergy or superiority of the combination over either component alone. Although the fixed-dose formulation has been shown to be bioequivalent to separate administration without a pharmacokinetic interaction [17], pharmacological interaction was not an outcome of the present study.

The clinical relevance of the present findings is best interpreted in terms of PLSP occurrence. During T_24h_, PLSP occurred in 39.7% of patients in the control group and 15.5% in the study group, corresponding to an absolute risk reduction of approximately 24 percentage points and a number needed to treat of approximately four. By contrast, maximum shoulder-pain intensity among patients who developed PLSP did not significantly differ between groups. The lower proportion of patients requiring rescue analgesia in the study group provides additional evidence of an early postoperative analgesic benefit. Abdominal-pain scores were also lower during TPACU and T_24h_, suggesting that the analgesic effect was not confined to PLSP. However, abdominal pain was not prospectively separated into incisional and visceral components; consequently, the relative contribution of the intervention to these different sources of pain could not be determined.

The reduction in rescue analgesic use should also be interpreted in the context of the overall treatment strategy. Fewer patients in the study group required ketorolac or fentanyl during the early postoperative period, which may reduce exposure to additional analgesic medications. However, some patients who received the fixed-dose paracetamol–ibuprofen combination subsequently received ketorolac as rescue analgesia, resulting in possible sequential exposure to different NSAIDs. Furthermore, prophylactic administration exposes all treated patients to medication irrespective of whether substantial postoperative pain would otherwise develop. Because this study was not designed as a formal safety or pharmacoeconomic comparison, the overall risk–benefit balance and cost-effectiveness of this strategy cannot be determined from the present findings.

Despite the lower proportion of patients requiring rescue analgesia, PONV did not differ significantly between groups. This finding should be interpreted cautiously because PONV is multifactorial and all patients received prophylactic ramosetron, which may have reduced overall event rates. Dexamethasone was also used as first-line rescue treatment despite its relatively delayed onset of action. In addition, the study was not specifically powered to detect differences in PONV. Accordingly, the absence of a significant between-group difference should not be interpreted as evidence of equivalence with respect to this outcome.

Several limitations should be considered. First, postoperative pain was assessed at rest and summarised within predefined postoperative intervals, whereas movement-evoked pain was not evaluated. Temporal variation in the onset and intensity of PLSP may therefore not have been fully captured. Second, although abdominal and shoulder pain were assessed separately, abdominal pain was not further classified into incisional and visceral components. Third, the indication for rescue analgesia was not recorded separately for abdominal and shoulder pain, and repeated rescue doses after PACU discharge and the exact time to first rescue analgesia were not recorded sufficiently consistently to permit reliable analysis of cumulative consumption or time-to-rescue. Fourth, this was a single-centre study with a modest sample size and a highly selected population restricted to ASA physical status I patients with a BMI < 30 kg/m^2^; patients with relevant contraindications to paracetamol or NSAIDs and those receiving regular paracetamol or NSAID therapy were also excluded. Therefore, the findings may not be generalisable to broader populations undergoing LC, including older patients, patients with obesity or comorbidities, and those at greater risk of NSAID-related adverse effects. Fifth, the absence of separate paracetamol, ibuprofen, and post-incisional comparator groups precludes conclusions regarding pharmacological synergy or a timing-dependent pre-emptive effect. Sixth, routine prophylactic ketorolac was not included in the perioperative analgesic regimen, which may differ from contemporary practice in which baseline multimodal analgesia is routinely used. Finally, follow-up was limited to 48 h, and systematic surveillance for treatment-emergent adverse events was not a prespecified study outcome. Therefore, no conclusions regarding comparative safety can be drawn.

In conclusion, pre-incisional intravenous administration of a fixed-dose paracetamol–ibuprofen combination was associated with a lower incidence of PLSP and a lower proportion of patients requiring rescue analgesia during the first 24 h after LC. However, among patients who developed PLSP, shoulder-pain severity did not differ significantly between the groups. Interpretation of both efficacy and safety should also consider that some patients in the intervention group subsequently received ketorolac as rescue analgesia.

## Figures and Tables

**Table 1:** Demographic and perioperative characteristics.

	Control Group (*n* = 58)	Study Group (*n* = 58)	*p* Value
Gender (F/M)	25/33	23/35	0.851
Age (years)	52 [40–58]	52 [40–60]	0.502
Height (cm)	158.4 ± 8.2	157.6 ± 7.4	0.340
Weight (kg)	63.9 ± 8.5	62.7 ± 9.6	0.470
Hx of motion sickness	5/58	6/58	1.000
Hx of PONV	9/58	8/58	1.000
Anaesthesia time (min)	85 [75–87]	86 [77–99]	0.116
Operation time (min)	72 [62–80]	71 [64–78]	0.135

Data are presented as number of patients, median [interquartile range], or mean ± standard deviation. Abbreviations: F, female; M, male; Hx, history; PONV, postoperative nausea and vomiting.

**Table 2:** Postoperative profiles.

			Control Group (*n* = 58)	Study Group (*n* = 58)	Effect Estimate (95% CI)	*p* Value
Abdominal pain (incidence/VAS: 0–100)						
	T_PACU_		58/58, [40 (30–50)]	58/58, [30 (20–40)]		-/<0.001
	T_24h_		58/58, [30 (30–40)]	58/58, [20 (20–30)]		-/<0.001
	T_48h_		58/58, [20 (10–20)]	58/58, [20 (20–20)]		-/0.324
PLSP (incidence)						
	T_PACU_		16/58 (27.6%)	5/58 (8.6%)	ARR 19.0% (5.4–32.5); RR 0.31 (0.12–0.80)	0.014
	T_24h_		23/58 (39.7%)	9/58 (15.5%)	ARR 24.1% (8.5–39.8); RR 0.39 (0.20–0.77)	0.006
	T_48h_		0/58	0/58		-
Shoulder-pain VAS (VAS: 0–100)						
	T_PACU_		30 [20–30]	30 [25–35]		0.577
	T_24h_		20 [20–30]	20 [20–30]		0.426
	T_48h_		-	-		-
Incidence of rescue for postoperative pain						
	T_PACU_					
		Ketorolac	36/58 (62.1%)	17/58 (29.3%)	ARR 32.8% (15.6–49.9); RR 0.47 (0.30–0.74)	<0.001
		Fentanyl	26/58 (44.8%)	9/58 (15.5%)	ARR 29.3% (13.5–45.1); RR 0.35 (0.18–0.67)	<0.001
	T_24h_					
		Ketorolac	20/58 (34.5%)	5/58 (8.6%)	ARR 25.9% (11.7–40.1); RR 0.25 (0.10–0.62)	<0.001
		Fentanyl	14/58 (24.1%)	3/58 (5.2%)	ARR 19.0% (6.6–31.4); RR 0.21 (0.07–0.71)	0.007
	T_48h_					
		Ketorolac	5/58 (8.6%)	2/58 (3.4%)	ARR 5.2% (−3.4–13.8); RR 0.40 (0.08–1.98)	0.438
		Fentanyl	0/58	0/58		-
PONV incidence/scale (0–3)						
	T_PACU_		17/58, [0 (0–1)]	12/58, [0 (0–0)]		0.391/0.378
	T_24h_		15/58, [0 (0–1)]	9/58, [0 (0–0)]		0.251/0.297
	T_48h_		6/58, [0 (0–0)]	5/58, [0 (0–0)]		0.743/0.752
Rhodes index (0–32)						
	T_24h_		0 [0–3]	0 [0–0]		0.263
	T_48h_		0 [0–0]	0 [0–0]		0.736
Incidence of rescue for PONV						
	T_PACU_					
		Dexamethasone	16/58	12/58		0.498
		Metoclopramide	9/58	6/58		0.608
	T_24h_					
		Ramosetron	5/58	6/58		0.856
	T_48h_					
		Ramosetron	3/58	5/58		0.707

Data are presented as number of patients, number/total number (%), or median [interquartile range], as appropriate. Shoulder-pain VAS values are presented only for patients who developed PLSP during the corresponding interval (T_PACU_: control *n* = 16, study *n* = 5; T_24h_: control *n* = 23, study *n* = 9). This symptomatic-patient analysis was performed as a post hoc exploratory reanalysis of the prespecified secondary outcome. No patient developed PLSP during T_48h_; therefore, shoulder-pain VAS was not applicable at this interval. Absolute risk reduction (ARR) was calculated as the event rate in the control group minus that in the study group. Relative risk (RR) was calculated as the event rate in the study group divided by that in the control group. For analysis, “incidence of rescue for postoperative pain” was defined as at least one administration during each interval. Abbreviations: ARR, absolute risk reduction; RR, relative risk; CI, confidence interval; VAS, visual analogue scale; PLSP, post-laparoscopic shoulder pain; PACU, post-anaesthetic care unit; PONV, postoperative nausea and vomiting.

## Data Availability

The raw data supporting the conclusions of this article will be made available by the authors on request.

## Supplementary Materials

The following supporting information can be downloaded at https://www.mdpi.com/article/10.3390/jcm15187249/s1, Figure S1: Distribution of repeated postoperative outcome measurements; Figure S2: Changes in intraoperative haemodynamic variables over time; CONSORT 2025 checklist.
