# Supplementary material for: Effect of Pre-Incisional Intravenous Paracetamol–Ibuprofen on Post-Laparoscopic Shoulder Pain After Laparoscopic Cholecystectomy: A Double-Blind Randomised Controlled Trial
Source: J Clin Med. 2026 Sep 18;15(18):7249. doi: 10.3390/jcm15187249 (PMC13607028; doi:10.3390/jcm15187249)
Supplement: Supplementary file 1 [file jcm-15-07249-s001.zip › Figure S1.pdf]

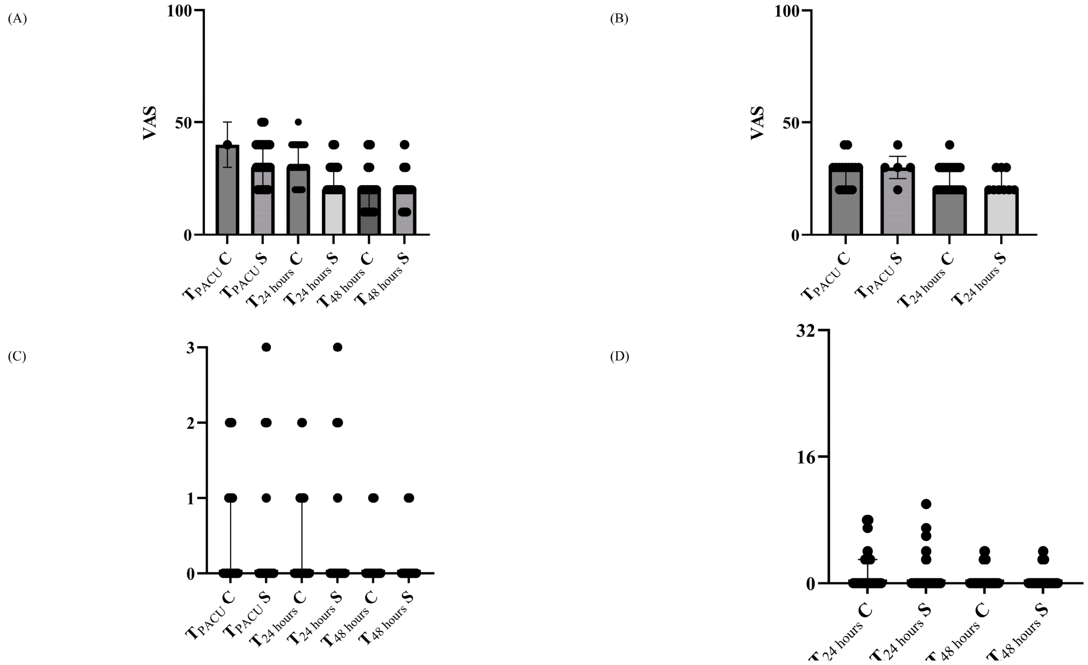

Figure S1. Distribution of repeated postoperative outcome measurements.

(A) Abdominal-pain VAS at T_{PACU}, $T_{24\ hours}$, and $T_{48\ hours}$. (B) Shoulder-pain VAS among patients who developed PLSP at T_{PACU} and $T_{24\ hours}$; no patient developed PLSP during $T_{48\ hours}$. (C) PONV severity scale at T_{PACU}, $T_{24\ hours}$, and $T_{48\ hours}$. (D) Rhodes index at $T_{24\ hours}$ and $T_{48\ hours}$. Data are shown as individual values with median and interquartile range. C, control group; S, study group.
