# Supplementary material for: Effect of Pre-Incisional Intravenous Paracetamol–Ibuprofen on Post-Laparoscopic Shoulder Pain After Laparoscopic Cholecystectomy: A Double-Blind Randomised Controlled Trial
Source: J Clin Med. 2026 Sep 18;15(18):7249. doi: 10.3390/jcm15187249 (PMC13607028; doi:10.3390/jcm15187249)
Supplement: Supplementary file 1 [file jcm-15-07249-s001.zip › Figure S2.pdf]

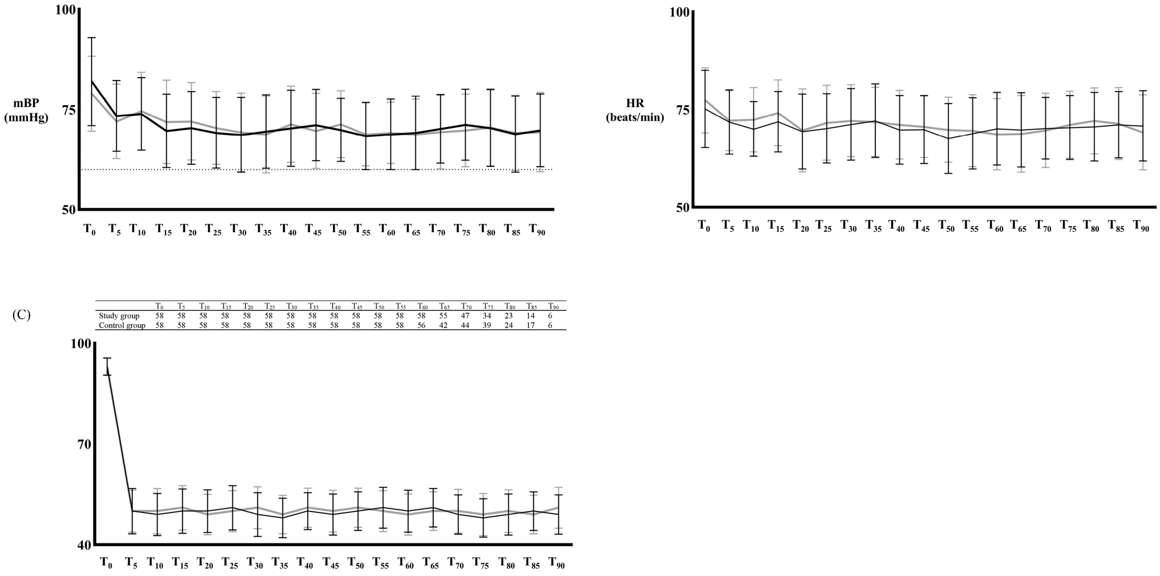

(A)

	T_0	T_5	T_{10}	T_{15}	T_{20}	T_{25}	T_{30}	T_{35}	T_{40}	T_{45}	T_{50}	T_{55}	T_{60}	T_{65}	T_{70}	T_{75}	T_{80}	T_{85}	T_{90}
Study group	58	58	58	58	58	58	58	58	58	58	58	58	58	55	47	34	23	14	6
Control group	58	58	58	58	58	58	58	58	58	58	58	58	56	42	44	39	24	17	6

(B)

	T_0	T_5	T_{10}	T_{15}	T_{20}	T_{25}	T_{30}	T_{35}	T_{40}	T_{45}	T_{50}	T_{55}	T_{60}	T_{65}	T_{70}	T_{75}	T_{80}	T_{85}	T_{90}
Study group	58	58	58	58	58	58	58	58	58	58	58	58	58	55	47	34	23	14	6
Control group	58	58	58	58	58	58	58	58	58	58	58	58	56	42	44	39	24	17	6

(C)

	T_0	T_5	T_{10}	T_{15}	T_{20}	T_{25}	T_{30}	T_{35}	T_{40}	T_{45}	T_{50}	T_{55}	T_{60}	T_{65}	T_{70}	T_{75}	T_{80}	T_{85}	T_{90}
Study group	58	58	58	58	58	58	58	58	58	58	58	58	58	55	47	34	23	14	6
Control group	58	58	58	58	58	58	58	58	58	58	58	58	56	42	44	39	24	17	6

Figure S2. Changes in intraoperative haemodynamic variables over time. (A) Mean blood pressure (mBP), (B) heart rate (HR), and (C) bispectral index (BIS). Data are presented as mean ± standard deviation. Grey and black lines represent the study and control groups, respectively. The numbers above each panel indicate the number of patients with available measurements at each time point. The dotted horizontal line in panel A indicates an mBP of 60 mmHg. Abbreviation: Tx, time from baseline (T0), where x represents minutes.
